# Endoscopic Third Ventriculostomy in Hydrocephalus Due to Complex Congenital Heart Disease: A Case Report

**DOI:** 10.7759/cureus.91062

**Published:** 2025-08-26

**Authors:** AJF Da Silva

**Affiliations:** 1 Pediatric Neurosurgery Division, Santa Mônica Teaching Maternity - Alagoas State University of Health Sciences and General State Hospital, Maceió, BRA

**Keywords:** central nervous system, congenital, heart, hydrocephalus, ventriculostomy

## Abstract

Congenital heart disease may be associated with extracardiac abnormalities, such as those of the central nervous system. We present the case of a child with complex congenital heart disease associated with obstructive hydrocephalus treated with endoscopic third ventriculostomy (ETV). A 10-month-old child presented with atrial and ventricular septal defects, a double-outlet right ventricle (DORV) with transposition of the great vessels, a dysplastic pulmonary valve, significant pulmonary stenosis, and patent ductus arteriosus on two-dimensional echocardiography done in an outpatient clinic. Furthermore, computed tomography of the skull showed ventricular dilatation with signs of ependymal transudation. The hydrocephalus was treated by ETV with good results. Hydrocephalus is one of the most common malformations of the central nervous system. Further studies are needed to further elucidate the pathophysiology of hydrocephalus, the best time to treat it, and whether ventriculoperitoneal shunt or ETV is the best choice of treatment.

## Introduction

Congenital heart disease (CHD) is defined as a substantial structural defect of the heart or the great intrathoracic vessels, which can have notable functional repercussions [1]. CHD accounts for 1% of all congenital malformations, and approximately 10% of infant deaths are due to congenital malformations [2]. The association with extracardiac abnormalities, for example, those of the central nervous system, determines a greater risk of morbidity and mortality [3]. The incidence of congenital hydrocephalus (CHC) differs worldwide. However, it falls in the range between 2.2 and 18 per 10,000 live births. Among different types of hydrocephalus, CHC accounts for around 50% [4]. When CHC is associated with CHD, there are two treatment options: extracranial shunts and endoscopic third ventriculostomy (EVT). The use of shunts can involve longer anesthesia time, increased patient handling during surgery, and a higher risk of infection. With EVT, when in experienced hands, the surgical procedure is performed in less time, thus reducing the use of drugs during anesthesia in this type of patient [5]. This study presents the case of a child with CHD associated with obstructive hydrocephalus who was treated with ETV.

## Case presentation

**Figure 1:**
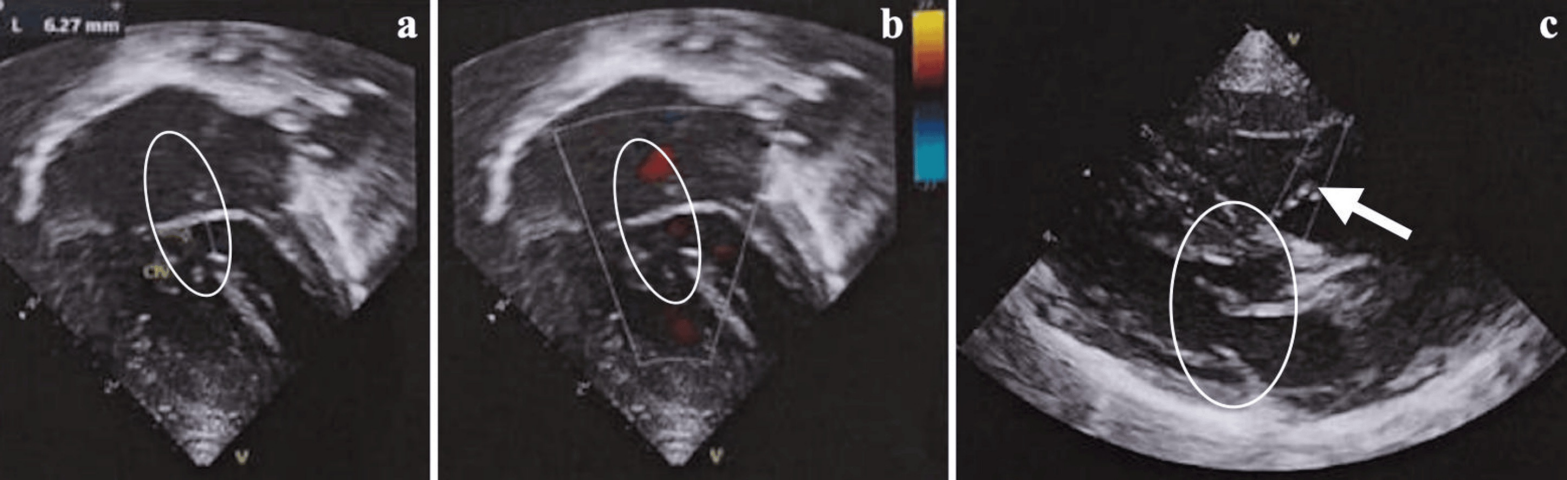
Two-dimensional echocardiography (a, b) Atrial and ventricular septal defect (circle). (c) Double-outlet right ventricle with transposition of the great vessels (white arrow). Dysplastic pulmonary valve, significant pulmonary stenosis, and patent ductus arteriosus (circle).

**Figure 2:**
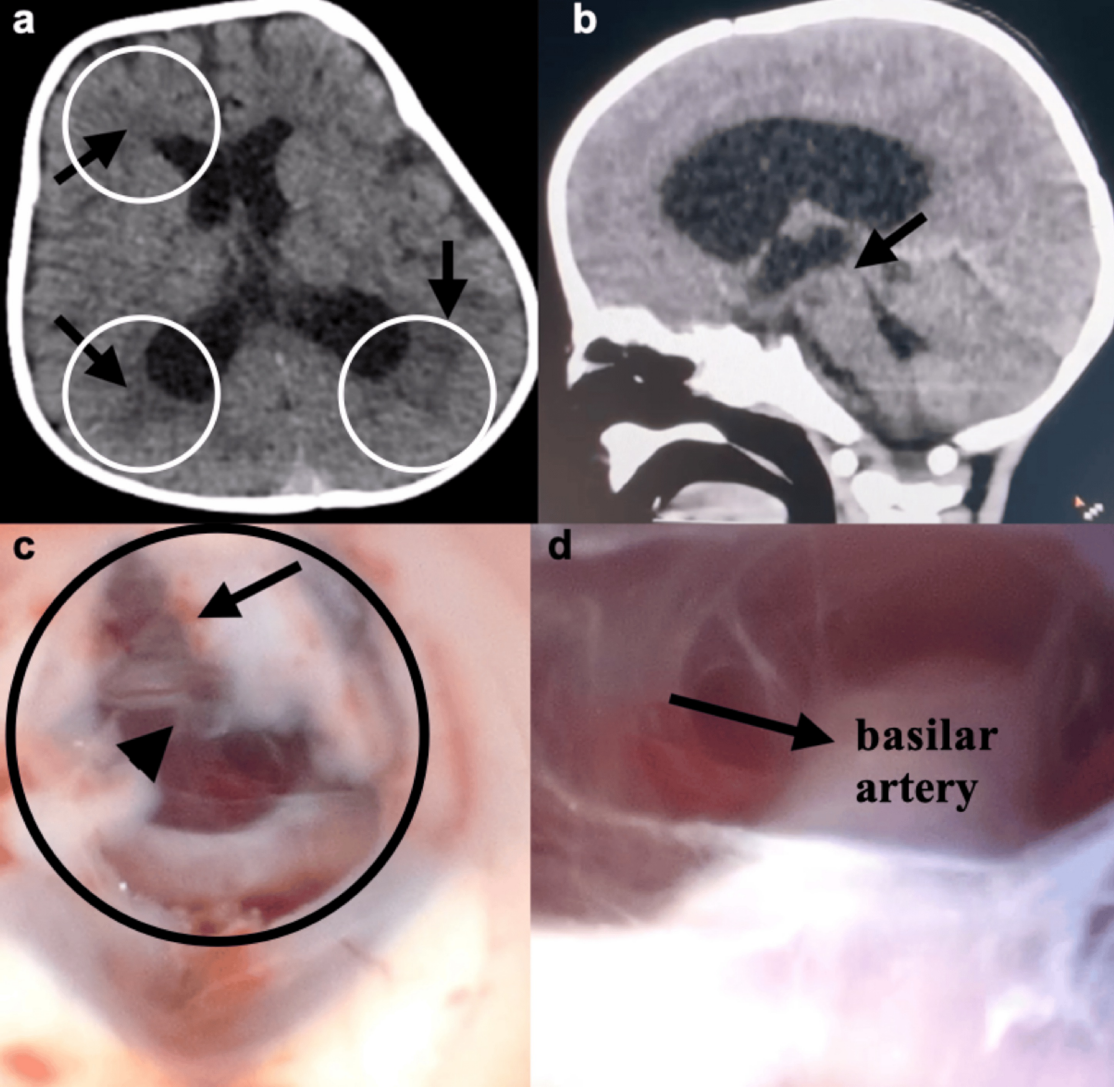
Computed tomography (CT) and endoscopic view (a) Axial CT scan of the skull showing hypertensive hydrocephalus with signs of ependymal transudation (black arrows). (b) Sagittal CT scan of the skull showing dilatation of the lateral ventricles and third ventricle with possible stenosis of the aqueduct of Sylvius (black arrow). (c) In the endoscopic view, it shows the tuber cinereum (black circle) with the first (black arrow) and second (black arrowhead) Liliequist membrane. (d) In the endoscopic view showing the prepontine cistern with the basilar artery (black arrow).

A 10-month-old infant was referred for neurosurgical assessment due to seizures and hypertensive hydrocephalus. This was a normal birth; the infant was full term, and the mother had no prenatal care and, therefore, did not know of any problems during the pregnancy. On physical examination, the infant was awake and active despite respiratory discomfort; oxygen dependency; baseline saturation of 75%-80%; cyanosis of the extremities; and a wide, bulging, and tense anterior fontanelle. Two-dimensional echocardiography revealed atrial and ventricular septal defect (Figures 1a-1b) with predominance of right-to-left shunt, double-outlet right ventricle with transposition of the great vessels (Figure 1c), dysplastic pulmonary valve, significant pulmonary stenosis, and patent ductus arteriosus (Figure 1c). Computed tomography of the skull showed ventricular dilatation with signs of ependymal transudation (Figures 2a-2b). Considering the hypertensive hydrocephalus, the decision to perform a neuroendoscopic procedure, namely ETV, was made. The surgery consists of making an opening in the floor of the third ventricle (in the tuber cinereum) to establish communication with the prepontine cistern (Figures 2c-2d). Sevoflurane was used as an inhalational anesthetic, and ketamine (2 mg/kg) as an intravenous anesthetic to maintain or increase systemic vascular resistance (SVR). There were no significant hemodynamic changes, and the surgical procedure was completed in less than one hour. The child is progressing relatively well neurologically, with no seizures.

## Discussion

Genetic or extracardiac abnormalities account for 22% of CHDs [6]. Central nervous system abnormalities account for 10%-75% of cases. Patent ductus arteriosus, complex ventricular wall defects, hypoplastic left heart, or pulmonary stenosis have been associated with a higher incidence of central nervous system disorders [7]. As in the case described herein, the presence of cardiac alterations (patent ductus arteriosus, ventricular wall defect, pulmonary stenosis) is related to central nervous system disorders.

The most common malformations of the central nervous system are microcephaly, hydrocephalus, agenesis of the corpus callosum, myelomeningocele, encephalocele, agenesis of the optic nerve, Dandy-Walker malformation, lissencephaly, and holoprosencephaly [2]. As in the case reported here, the malformation of the central nervous system is hydrocephalus.

The association of symptomatic hydrocephalus and CHD results in increased mortality and severe developmental delay [6]. Studies have shown that cerebrospinal fluid moves in the subarachnoid space in synchrony with the cardiac cycle. Hydrocephalus is not a local brain phenomenon, as the blood flow in the brain is part of the general cardiovascular blood supply response. When a cardiovascular disease reduces blood volume, the brain is left without a force vector to oppose the expanding ventricular system. Therefore, hydrocephalus is not always non-communicating as it can be the result of an imbalance in the distribution of cardiac output, leading to chronic communicating hydrocephalus [8]. Other possible causes of hydrocephalus in the case described may be related to hemorrhage and infection. However, obstructive hydrocephalus due to aqueductal stenosis was confirmed. In such a case, two forms of treatment can be used: placement of a ventriculoperitoneal shunt or ETV. The advantage of EVT is that it offers better results in terms of infection risk and failure rate [5]. However, the rate of secondary surgery is significantly higher for pediatric patients, especially those less than one year of age [9]. As there are not enough studies comparing the two techniques in children with CHD, we decided to use the neuroendoscopic procedure.

## Conclusions

CHD can be associated with genetic or extracardiac abnormalities, including the central nervous system, thus increasing morbidity and mortality. In this report, a case of complex CHD associated with hydrocephalus is described. The patient, even though he was under one year old, was treated with ETV with good clinical progress. EVT proved to be a safe method, performed in less than an hour and with a lower risk of infection. Further studies are needed to better clarify the pathophysiology of this hydrocephalus, the best time to treat it, and the best choice of treatment: ventriculoperitoneal shunt or ETV.
